# Interactions between Nod-Like Receptors and Intestinal Bacteria

**DOI:** 10.3389/fimmu.2013.00462

**Published:** 2013-12-17

**Authors:** Marcel R. de Zoete, Richard A. Flavell

**Affiliations:** ^1^Department of Immunobiology, Yale University School of Medicine, New Haven, CT, USA; ^2^Howard Hughes Medical Institute, Yale University, New Haven, CT, USA

**Keywords:** nod-like receptors, microbiota, inflammasome, intestine, pathogen

## Abstract

Nucleotide oligomerization domain (Nod)-like Receptors (NLRs) are cytosolic sensors that mediate the activation of Caspase-1 and the subsequent processing and secretion of the pro-inflammatory cytokines IL-1β and IL-18, as well as an inflammatory cell death termed pyroptosis. While a multitude of bacteria have been shown to activate one or more NLRs under *in vitro* conditions, the exact impact of NLR activation during the course of colonization, both of pathogenic and commensal nature, is less understood. In this review, we will focus on the role of intestinal NLRs during the various stages of infection with common gastrointestinal bacterial pathogens, as well as NLR function in controlling and shaping the microbiota.

## Introduction

The human body lives in symbiosis with trillions of microbial cells, collectively called the microbiota, with the vast majority of these microbes being bacteria that inhabit the gastrointestinal tract (1). This symbiosis begins with colonization of the gastrointestinal tract at birth and then is sustained throughout life by environmental exposures (2). Occasionally this microbial symbiosis is challenged by invading bacterial pathogens, which perturb the microbial ecosystem and cause disease.

Our ability to harbor trillions of bacteria within our intestines relies on the maintenance of a safe distance between these bacteria and the single layer of intestinal epithelial cells. Crucial protective mechanisms have evolved to help ensure host-bacteria mutualism. A major barrier bacteria encounter in the intestine is formed by the mucus layer, a dense network of glycoproteins that most bacteria are unable to breach (3). To further aid the barrier function of the mucus layer, intestinal cells also secrete an array of antimicrobial proteins, like antimicrobial peptides, lectins, and lysozymes. Furthermore, secreted IgA specifically targets bacteria for immune exclusion (4).

At the cellular level, sensing systems continuously scan for bacteria that are able to actively surpass the mucus layer and attach to and/or invade the epithelium. Two major receptor families that detect microbes are the Toll-like Receptors (TLRs), which control the extracellular compartment, and Nod-like Receptors (NLRs), which sense the presence of intracellular microbes (5). NLRs are crucial for fighting and resolving infections as many pathogenic bacteria (and under certain conditions also members of the commensal microbiota) attempt to exploit and enter the cytosol for nutrients and to escape extracellular threats (6). Here, we provide an overview of the role of NLRs in protection against intestinal pathogenic bacteria and control of the intestinal microbiota.

## Intestinal NLRs

Nod-like receptors generally consist of a ligand-sensing domain in the form of a Leucine Rich Repeat (LRR) domain, a central ATP binding domain, and a signaling domain (often in the form of a CARD or Pyrin domain) and are categorized by their domain structure. While NLRs are expressed widely in a variety of tissues in humans and mice, we will focus in this review on those that were shown to function in the defense against bacteria in the intestine. While Absent In Melanoma 2 (AIM2) theoretically is not part of the NLR family, we have included it here for completeness.

### NOD1 and NOD2 (NLRC1, NLRC2)

The pattern recognition receptors NOD1 and NOD2 are amongst the best-studied NLRs, and their ligands are well defined. Both NOD1 and NOD2 sense cytosolic bacterial peptidoglycan fragments with high specificity: NOD1 is activated by d-glutamyl-meso-diaminopimelic acid (DAP) containing peptidoglycan fragments, which are mainly found in Gram-negative bacteria (7), whereas NOD2 was shown to bind and responds to muramyl dipeptide (MDP), found in all bacteria (8). Despite the presence of N-terminal CARD domains, NOD1 and NOD2 are non-inflammasome forming NLRs and do not seem to directly activate Caspase-1. Instead, after ligand binding the CARD domain of NOD1 and NOD2 interacts with the signaling kinase RIP2 (RIPK2, RICK) that initiates a signaling cascade resulting in NF-κB activation, as well as the activation of ERK, p38 and mitogen-activated protein kinases (MAPKs) (9, 10). These signaling pathways result in the expression of a variety of pro-inflammatory cytokines and chemokines, as well as the production of reactive oxygen species. While NOD2 expression is more restricted, both NODs are expressed in macrophages, dendritic cells, Paneth cells, and intestinal epithelial cells, making them highly suited to sense infections throughout the intestinal tract (10). In recent years, several layers of complexity were added onto the basic mechanism of NOD1 and NOD2 sensing and signaling. For instance, NOD2 was shown to interact with NLRP1, NLRP3, and NLRP12 (11), NOD1 and NOD2 were found to play a role in autophagy (12), and NOD1 senses the modification of small rho GTPases injected by *Salmonella* during infection (13).

### NLRC4

NLRC4 is an N-terminal CARD domain containing NLR. The elucidation of the NLRC4 crystal structure has revealed that, under resting conditions, NLRC4 resides in a closed monomeric form, kept in place by an ADP-dependent autoinhibitory mechanism involving multiple domains including the LRR (14). Ligand binding is proposed to induce “opening” of the structure, the exchange of ADP for ATP, and subsequent NLRC4 oligomerization. Phosphorylation of a conserved serine residue proximal to the LRR was shown to be required for NLRC4 inflammasome activation in macrophages, although the exact role in this process requires further investigation (15). NLRC4 responds to attaching or invading pathogens by sensing their bacterial secretion systems. So far, two bacterial ligands are well defined: flagellin, which is co-secreted with virulence factors either through type III or type IV secretion systems (T3SS and T4SS, respectively) (16–18), and PrgJ, a structural component of the type III secretion system that leaks or is secreted into the host cytosol (19). Within the cytosol, flagellin and PrgJ bind to the adapter proteins NLR family, apoptosis inhibitory protein (NAIP) 2 and NAIP5, respectively (20, 21), which subsequently bind NLRC4 to initiate its oligomerization into a ring-like inflammasome that recruits the adapter protein apoptosis-associated speck-like protein containing a CARD (ASC) (containing both a Pyrin and CARD domain) and Caspase-1 (22). This complex then processes the pro-inflammatory cytokines pro-IL-1β and pro-IL-18, and induces pyroptosis, an inflammatory form of cell death. Interestingly, unlike mice, humans have only one NAIP protein, which is unresponsive to both flagellin or basal rod protein but instead binds the conserved T3SS needle protein to activate NLRC4 (20).

### NLRP3

While NLRP3 is probably the best studied of the NLRs, the mechanism of receptor activation remains relatively unclear. NLRP3, or cryopyrin, was originally shown to play a key role in a collection of autoinflammatory disorders collectively termed cryopyrin-associated periodic syndromes, which all share mutations in NLRP3 that lead to inappropriate IL-1β-mediated inflammatory responses (23). NLRP3 was subsequently found to “sense” a long list of ligands or stimuli, including ATP, pore-forming toxins, particulates like asbestos and silica, bacteria, viral, and fungal infections (24). Initially, three main theories of the activation of NLRP3 were proposed: potassium efflux, lysosomal rupture and subsequent cleavage by released Cathepsin, and ROS production. Several “second generation” unifying NLRP3 ligands were proposed to combine the three, including oxidized mitochondrial DNA released into the cytosol following mitochondrial damage (25); thioredoxin-interacting protein (26), calcium mobilization (27), mitochondrial cardiolipin (28), and changes in cell volume (29). While most of these NLRP3 ligands were recently shown to lead to potassium efflux might, suggesting this to be the common trigger in the end (30), NLRP3 activation remains enigmatic; structural studies similar to those done for NLRC4 might eventually elucidate the elusive NLRP3 ligand.

A new chapter for NLRP3 has been opened through the elucidation of the non-canonical inflammasome pathway. Due to the (re)discovery of the presence of a mutated, non-functional Caspase-11 in the original Caspase-1-deficient mouse, a role for Caspase-11 was found in NLRP3-inflammasome activation by Gram-negative bacteria (31). After prolonged (~17h) stimulation of bone-marrow macrophages with bacteria, Caspase-11 was shown to be activated, leading to cell death and NLRP3/ASC/Caspase-1-dependent IL-1β and IL-18 secretion. It was subsequently shown that the TLR4-TRIF-Type I Interferon pathway was required to induce high levels of Caspase-11 transcription needed for non-canonical inflammasome activation (32). However, it recently was shown that intracellular LPS serves as a ligand able to activate the non-canonical inflammasome pathway, independently of increasing levels of Caspase-11 caused by Type I Interferon (33). Three major questions regarding non-canonical inflammasome activation remain currently unanswered: what is the receptor that senses intracellular LPS or potentially other ligand (presumably a CARD-containing NLR), how does this complex feed into the NLRP3 inflammasome (Caspase-11-dependent pyroptosis resulting in potassium efflux?), and is Caspase-11 activated by any additional receptors?

### NLRP6

NLRP6 falls within the group of NLRs that was initially found to induce NF-κB and Caspase-1 activation during overexpression in transfected tissue culture cells (34). In this system, human NLRP6 was also shown to form punctate structures in the cytoplasm, but only in the presence of ASC, suggesting the ability of NLRP6 to form inflammasomes or inflammasome-like structures. Unlike in humans, where NLRP6 is not highly or widely expressed, mice exhibit high NLRP6 expression throughout the intestine, kidneys, and liver (35, 36), which can be regulated by stress factors (37). Mechanistically, NLRP6 was shown to be a negative regulator of NF-κB and MAPK in cultured bone-marrow macrophages from NLRP6-deficient mice (38), which is the opposite of what was initially observed in overexpression studies. NLRP6 function as a negative regulator of NF-κB and MAPK might play a role in the increased intestinal tissue proliferation and inflammation observed in NLRP6-deficient mice (39, 40). Furthermore, NLRP6 was shown to be involved in the production of Caspase-1 dependent IL-18 (36), again suggesting the ability of NLRP6 to form an inflammasome.

### NLRP12

Since its identification, NLRP12 has been assigned a number of different functions. Like NLRP6, NLRP12 was originally described to induce NF-κB and Caspase-1 activation when co-expressed with ASC (41). In contrast, without ASC co-expression, NLRP12 overexpression reduced non-canonical NF-κB activation and enhanced the expression of non-classical and classical MHC class I genes (42–44). NLRP12-deficient mice were also reported to have defective dendritic cell and neutrophil responses to chemokines and subsequent defective dendritic cell migration to draining lymph nodes (45). In addition, NLRP12-deficiency led to enhanced colon inflammation and colorectal cancer development due to increased (non-canonical) NF-κB and ERK activation (46, 47), similar to what was observed previously for NLRP6.

### AIM2

AIM2 was originally identified in humans as an interferon-inducible, putative tumor suppressor protein (48), but subsequently found to sense cytoplasmic double stranded (ds) DNA, form an inflammasome complex together with ASC and Caspase-1, and trigger the processing of pro-IL-1β and pro-IL-18 (49–52). Like NLRP receptors, AIM2 contains an N-terminal signaling Pyrin domain; however, the C-terminal consists of a DNA-binding HIN200 domain. AIM2 is able to sense the presence of cytosolic non-sequence-specific dsDNA of both viral and bacterial origin. Bacterial DNA enters the cytosol mainly by a passive process following bacterial lysis, for instance during rapid cytosolic bacterial replication or after intracellular bacteria-containing vesicles are compromised, but appears to be always preceded by bacterial invasion into the host cell, making AIM2 a specific sensor for intracellular bacteria and viruses (53–55). In a mechanism similar to what was observed for NLRC4, the HIN200 domain functions as a negative regulator of the signaling Pyrin domain. Non-sequence-specific binding of dsDNA releases this inhibition, liberating the Pyrin domain to recruit ASC and Caspase-1, and form an inflammasome surrounding the released bacterial or viral DNA (56, 57).

## Enteric Pathogens and Their Interactions with NLRs

Foodborne gastrointestinal pathogens are a major cause of bacterial infections in humans (58). Studies on these pathogens, both in the human host and in various murine models, have provided great insights into microbial virulence mechanisms as well as the immunological defense strategies of the host. For NLRs, pathogen-host interactions have been of great value for the elucidation of the different functions of this family of innate sensors, both in *in vitro* and *in vivo* model systems. Below (and summarized in Table 1), we provide an overview of the role of NLRs during infections with the most commonly studied bacterial enteric pathogens.

**Table 1:** **Role of NLRs in intestinal bacterial infections**.

Bacteria	Model	NLR	Mechanism of action	Reference
*S. typhimurium*	Systemic	NLRC4	Flagellin/T3SS-induced pyroptosis, IL-1β, and IL-18 production	(79–81)
		NLRP3	Caspase-1 activation	(79)
	Systemic, T3SS-1-independent	NOD1	Nitric oxide production in dendritic cells	(69)
	Systemic and colitis in Balb/c	NLRC4	IL-1β-mediated neutrophil recruitment	(84)
	Colitis	NLRC4	IL-1β and IL-18 production	(80)
		NOD1	NOD1-mediated detection of SipA	(70)
	Colitis, T3SS-1-independent	NOD1/2	Innate CD4^+^ T helper type 17 cell responses in the cecum	(74, 75)
	Systemic (intraperitoneal)	NLRP6	NLRP6-mediated negative regulation of NF-κB and MAPK activation	(38)
*C. rodentium*	Colitis	NOD2	NOD2-activation in stromal cells, CCL2/CCR2-dependent recruitment of inflammatory monocytes, IL-12-mediated bacterial clearance	(92)
		NOD1/2	IL-6-dependent IL-17 production in the cecum	(75)
		NLRC4	IL-1β and IL-18 production	(94)
		NLRP3	IL-1β and IL-18 production	(94)
*H. pylori*	Gastritis	NOD1	T4SS-mediated delivery of peptidoglycan, NF-κB-mediated inflammatory responses	(108)
		Unknown	IL-18-dependent IL-17 production, T-cell-mediated antibacterial responses	(110, 112)
		Unknown	IL-1β-dependent impaired bacterial clearance	(111, 113)
Microbiota	Colitis (DSS)	NLRP6	IL-18/CCL5 production, increased intestinal epithelial proliferation and tissue repair	(36, 40)
		NLRP3	Both increased and decreased susceptibility; microbiota-dependent?	(124, 125, 127, 128)
		NOD1/2	Induction of E-cadherin and RegIII-γ expression	(120)
	Colorectal cancer (DSS-AOM)	NLRP6	IL-18/CCL5/IL-6 mediated increased intestinal epithelial proliferation and tissue repair	(39, 40, 116)
		NLRP3	Caspase-1 activation	(126)
	Non-alcoholic fatty liver disease	NLRP3/6	IL-18-mediated control of microbiota	(117)

### Salmonella

In humans, infections with the Gram-negative bacterium *Salmonella enterica* (*S*. *enterica*) generally result in one of two distinct clinical phenotypes. *S. enterica* serovars Typhi and Paratyphi are the causative agents of Typhoid fever, a life-threatening disease characterized by systemic spread of ingested bacteria, high fever, and intestinal bleeding that results in 200,000–600,000 deaths worldwide each year (59). The more common non-typhoidal *S*. *enterica* serovars, like *S*. *typhimurium* and *S*. *enteritidis*, cause a self-limiting gastroenteritis characterized by (bloody) diarrhea, fever, abdominal cramps, and vomiting that usually lasts 4–7 days and affects over 90 million people worldwide each year (60).

Two different disease models in mice represent the two clinical manifestations of *Salmonella* infections. The classical murine model of systemic *S. typhimurium* infection induces a disease similar to Typhoid fever. In this model, orally administered salmonellae reach the distal ileum within hours of ingestion and, aided by flagella-mediated motility and chemotaxis (61), cross the mucus layer toward the epithelium. Here, the bacteria target the follicular-associated epithelium overlying the Peyer’s Patches, with a strong preference for the M-cells, as a main port of entry. To gain access into the host cell, *Salmonella* employs the first of two type III secretion systems (T3SS-1), which is only expressed during this initial phase of infection, and injects an array of effector proteins into the host’s cytosol that induce cytoskeletal rearrangements leading to bacterial invasion (62). Other effector proteins and the activation of pattern recognition receptors initiate inflammatory responses that attract neutrophils, monocytes, and macrophages. *Salmonella* replicates within the Peyer’s Patches and disseminates to mesenteric lymph nodes (MLN), liver, and spleen within infected monocytes and dendritic cells. In a T3SS-1-independent manner, a small proportion of the bacteria is also taken up passively from the lumen by CD11c^+^ CX3CR1^+^ dendritic cells and transported directly to the MLN, bypassing the Peyer’s Patches (63). To survive and replicate in the host’s cells, *Salmonella* resides within a vacuole, the *Salmonella*-containing Vacuole (SCV), whose integrity is maintained by the effector proteins secreted into the cytosol through a second T3SS (T3SS-2). During the later stages of disease, disseminated bacteria in the liver and spleen are found mostly within macrophages, in which they rapidly replicate. Continuous cycles of bacterial replication and dissemination eventually lead to bacteremia from which mice succumb after a week.

In the murine colitis model, infection with *Salmonella* is preceded by a single dose of streptomycin, which is believed to briefly reduce “colonization resistance” provided by the microbiota that occupies the more distal intestinal tract and strongly competes for nutrients. In this short window, *Salmonella* is able to gain a foothold in the cecum and colon, and replicates to high numbers within hours of infection, limiting the need for systemic spread (64). Colonization is accompanied by mucosal penetration of bacteria and the development of colitis, similar to the pathology seen in human infection with non-typhoidal serovars. For efficient colonization salmonellae still require T3SSs, which mediate invasion of enterocytes and induction of (local) inflammation. A reason for this was presented in an elegant study by Winter et al. which revealed that T3SS-induced inflammatory responses are actively exploited by *Salmonella* through the ability to utilize tetrathionate, formed in the intestine under inflammatory conditions, to successfully compete with the microbiota (65). In the colitis mouse model, *Salmonella* also exploits a T3SS-1-independent, “passive” route for uptake through CD11c^+^ CX3CR1^+^ dendritic cells. Similar to what is seen in the systemic model, these two invasion pathways act in concert, although T3SS-1-mediated invasion seems much more dominant. While bacteria are able to grow extensively in the distal intestine in the colitis model, substantial numbers of bacteria still continue to disseminate to liver and spleen, and infected mice usually die after 5–6 days.

As several phases of infection largely rely on cellular invasion, NLRs appear to be the ideal sensing mechanism for *Salmonella*. *In vitro, Salmonella* is sensed by NOD2 in cultured intestinal epithelial cells, which enables the control of intracellular bacteria though the induction of antimicrobial responses and autophagy (66–68), and by NOD1 in bone-marrow-derived dendritic cells, resulting in nitric oxide production (69). Interestingly, while *Salmonella* peptidoglycan may be involved in the activation of NOD1 and NOD2, it was recently shown that the modification of small Rho GTPases by the T3SS-1 effector SopE, which enables bacterial invasion, is a danger signal sensed by NOD1 (13). Presumably through a similar mechanism, another T3SS-1 effector SipA activated NOD1/NOD2-dependent NF-κB responses both *in vitro* and *in vivo* (70). In addition to NOD1 and NOD2, *Salmonella* is efficiently sensed by NLRC4, both via flagellin and the basal rod protein PrgJ, which leads to rapid pyroptosis and secretion of IL-1β and IL-18 by cultured macrophages, dendritic cells and B-cells (16, 17, 19, 71, 72). These responses are solely dependent on T3SS-1, which is expressed only at the early logarithmic phase of bacterial cultures and is believed to represent the early phase in infection when *Salmonella* needs to invade host cells. The T3SS-2, expressed only at the late logarithmic phase, does not contribute to NLRC4 activation as flagellin expression is now repressed and the T3SS-2 apparatus is not recognized (19). Finally, *Salmonella* has been shown to activate the non-canonical NLRP3 inflammasome through Caspase-11 in macrophages (73).

*In vivo*, the role of NLRs during *Salmonella* infection has been rather difficult to define, mainly because of differences in experimental models (systemic versus colitis murine models), variations in growth phase of *Salmonella* at time of infection (T3SS-1-expressing versus T3SS-2-expressing conditions), different mouse intestinal microbiotas, and because of the redundancy in innate receptors. These issues are clearly demonstrated when studying NOD1 and NOD2. Both of these NLRs were shown to be dispensable during systemic infection (69). However, under T3SS-1-independent conditions, during which the bacteria “passively” cross the epithelial barrier through uptake and transport by dendritic cells, NOD1 deficiency led to higher bacterial loads and mortality. The authors show that NOD1-deficient CD11b^+^CD11c^+^ dendritic cells contain higher numbers of *Salmonella*, likely because of a diminished NOD1-mediated nitric oxide response. Similarly, during *Salmonella* colitis, NOD1/NOD2 double knockouts or RIP2 deficient mice exhibited reduced inflammation accompanied by increased mucosal colonization and reduced early IL-17 responses of innate CD4^+^ T helper type 17 cells in the lamina propria of the cecum, but again only when *Salmonella* was grown under T3SS-2-expressing conditions (74, 75); when T3SS-1 was expressed at the time of oral infection in this model, no differences as compared to wild-type mice were observed (76). These data suggest that only one of the two major entry pathways exploited by *Salmonella* is controlled by NOD1/NOD2 signaling. During “normal” infections, however, the T3SS-1-mediated invasion seems to outweigh the alternative invasion route, leaving NOD1 and NOD2 to play a non-significant role. Interestingly, NOD1/NOD2-activation by the T3SS-1 effector SipA was shown to lead to a higher gut inflammation score in the colitis model as compared to mice lacking the receptors (70). Combined, the above-mentioned studies suggest that NOD1 and NOD2 mediated detection of *Salmonella* plays a specific but minor role during salmonellosis. As a further complication, differences in microbiota from wild-type and knockout mice dramatically impact *Salmonella* susceptibility, as demonstrated by Kaiser et al.; when ”microbiota-matched” littermate controls were used (instead of independently bred or purchased wild-type mice) to test the role of RIP2 during *Salmonella* colitis, the initially observed difference in *Salmonella*-induced pathology was completely lost (64).

Caspase-1 has been shown in several publications to provide moderate protection against *Salmonella* infection. Without Caspase-1, mice succumb to bacteremia sooner and have higher bacterial loads in the MLN, liver and spleen in the typhoid model of infection, and more colitis accompanied by increased bacterial mucosal infiltration (77, 78). While NLRC4 appeared to be the main upstream candidate for caspase-1 activation, NLRC4-deficient mice showed only minor or no defects in bacterial control during infections (77, 79, 80). Two critical findings explained this “lack” of NLRC4 function *in vivo*; first, *Salmonella* actively evades recognition by NLRC4 by downregulating both T3SS-1 and flagellin as soon as the bacteria have invaded the host cell, and T3SS-2 is not recognized by NLRC4 (19, 81). The *in vivo* consequence of NLRC4-evasion, and thereby the role of NLRC4 in protection against invading pathogens, was elegantly shown by Miao et al.: when *Salmonella* was forced to continuously express flagellin, 100 times less bacteria were found in the spleen after 48 h during systemic infection. The increased control of bacterial spreading was attributed to NLRC4-mediated macrophage pyroptosis at peripheral sites, which resulted in release of the intracellular bacteria and subsequent clearance by infiltrating neutrophils (81).

NLR redundancy is a second reason why NLRC4-deficient mice do not phenocopy Caspase-1-deficient mice. Late logarithmic, non-T3SS-1 expressing salmonellae are able to activate the non-canonical Caspase-11/NLRP3 inflammasome (73). Similar to NLRC4, deficiency in only NLRP3 does not lead to differences in *Salmonella* infection. However, deletion of both NLRC4 and NLRP3 recapitulates the Caspase-1 phenotype completely, confirming a role for both NLRC4 and NLRP3 during *Salmonella* infection (79). This also demonstrates that, as was predicted by *in vitro* studies, NLRC4-evasion is not perfect. While pyroptosis has a clear impact on infection, the cytokines IL-1β and IL-18 appear to play minor roles in the control of the bacteria, since IL-1β and IL-18-deficent mice show little delay in bacteremia at 72 h (81, 82).

With the realization that the Caspase-1 KO was in fact a Caspase-1/Caspase-11 double knockout, and the elucidation of the role of Caspase-11 in non-canonical inflammasome activation, Caspase-11-deficient mice were predicted to result in more bacterial spread due to diminished control of infection. However, Caspase-11-deficient mice were indistinguishable from WT mice during *Salmonella* infection (79). Surprisingly, Caspase-1 single deficient mice had even higher numbers of bacteria in liver and spleen than the Caspase-1/Caspase-11-deficient mice, suggesting a protective role for Caspase-11 deficiency, but only in the context of Caspase-1-deficiency. A potential explanation for this may be that, while rapid Caspase-1-mediated pyroptosis clears bacteria, Caspase-11-mediated pyroptosis at later time points is actively used by *Salmonella* to escape the “full” macrophage after extensive replication. Indeed, Caspase-11 senses bacteria escaping from or leaking out of vacuoles into the cytoplasm (83). In the absence of Caspase-1, NLRC4 “evasion” by *Salmonella* is complete, resulting in uncontrolled replication until Caspase-11 is utilized to break out of the macrophage and invade new host cells. Why non-canonical Caspase-11-mediated pyroptosis, like NLRC4-activation accompanied by IL-1β and IL-18 secretion that induces local inflammation and attracts neutrophils, is less potent than Caspase-1-mediated pyroptosis in controlling *Salmonella* infection remains thus far unclear.

Unlike in C57BL/6 mice, in Balb/c mice NLRC4 appears to have a more prominent function in controlling *Salmonella* infection. In these mice, NLRC4-deficiency leads to more systemic bacterial dissemination and mortality, while less inflammation-induced pathology in the cecum was observed (84). It was subsequently shown that *Salmonella* specifically activates intestinal phagocytes that respond by producing IL-1β which triggered the upregulation of endothelial adhesion molecules. The basis of the interesting differential function of NLRC4 between C57BL/6 and Balb/c remains to be determined.

### Attaching and effacing enteric pathogens: *Citrobacter*, EPEC and EHEC

*Citrobacter rodentium* (*C*. *rodentium*), Enteropathogenic *Escherichia coli* (EPEC), and Enterohemorrhagic *Escherichia coli* (EHEC) are Gram-negative extracellular enteric pathogens that share a similar virulence strategy, termed attaching and effacing (A/E) (85, 86). EPEC and EHEC are human pathogens; EPEC is a major cause of diarrhea in young children, generally without major complications, while EHEC infections can vary greatly in severity, ranging from mild gastroenteritis to severe hemorrhagic colitis and hemolytic uremic syndrome. *C*. *rodentium* is a natural mouse pathogen resulting in self-limiting enteritis. While very little is known about activation of NLRs by EPEC and EHEC in humans, several studies have elucidated the role of such responses during infection of their murine counterpart, *C*. *rodentium*.

Within a couple of hours after oral infection of mice, *C*. *rodentium* reaches its initial site of infection, the lymphoid tissue in the cecum termed the cecal patch, where it reaches high density over the following 3 days (87). The cecal patch is structurally similar to the Peyer’s Patch and, because of their nature as antigen sampling hotspots with decreased mucus layer thickness and absence of microvilli, provide “easy access/entrance” for several intestinal pathogens (including *Salmonella*, as described above). From day 3 to 4, *C. rodentium* starts to spread throughout the distal colon (87). Mouse-adapted strains of *C*. *rodentium* largely skip the cecal patch phase and colonize the colon readily, suggesting that colonization of the cecal patch also serves as an adaptation phase to the mouse intestinal environment (88). Depending on the strain of *C. rodentium* used, bacterial numbers peak between day 5 and 14 with limited systemic spread to the MLN, liver, and spleen. The colonization then slowly diminishes until bacterial clearance from the cecum and subsequently the colon after 3–4 weeks post infection.

Upon reaching the cell surface of the cecum and colon, *C. rodentium* employs a T3SS which injects an array of virulence factors into the host’s cytosol that result in the attachment of the bacteria to the enterocytes and the accompanying local destruction of the brush border microvilli of the epithelium forming pedestal-like structures termed A/E lesions (86). Two of these virulence proteins are central for this virulence strategy: the adhesin Intimin expressed on the bacterial surface and the T3SS-injected Translocated Intimin Receptor (TIR), which provides a docking ligand for Intimin on the host epithelial surface (89, 90). The attachment of *C. rodentium*, in combination with the secretion of many additional virulence proteins, leads to colonic hyperplasia, observed readily during the peak of infection as larger intestinal crypt length and increased colon weight.

Several reports have shown that NOD1 and NOD2 are able to sense *C. rodentium* both *in vitro* and *in vivo* (75, 91, 92). In the absence of NOD2, *C. rodentium* reaches a higher intestinal abundance as compared to wild-type mice. At the early stages of infection, NOD2 signaling was shown to activate the CCL2/CCR2 axis that resulted in the recruitment of inflammatory monocytes to the site of infection, which initiated IL-12-mediated bacterial clearance. Interestingly, NOD2-activation took place in intestinal stromal cells and not immune cells. NOD2-deficiency led to lower inflammation at the early stages of infection, but more severe colitis later, as a result of reduced clearance and higher bacterial abundance in the intestine (92). In a different study, NOD1 and NOD2 had redundant roles in the protection against *C. rodentium* infection and mediated IL-6-dependent IL-17 production in the cecum at early time point (1–4 days after infection). The observed effect on infection was similar; lower initial inflammatory responses but increased levels of bacterial dissemination to the spleen in the second week of infection (75). NOD1/NOD2 signaling was shown to occur mostly in the radio-resistant compartment, but a role for stromal cells was not further investigated. Although it is expected that peptidoglycan is the major *C. rodentium*-derived ligand of NOD1 and NOD2, T3SS-injected effector proteins may play a role too, as was shown previously for the *Salmonella* effector protein SopE (13). Indeed EspT, which targets small GTPases to induce membrane rearrangement in a similar way as SopE, was shown to induce NF-κB, ERK1/2 and JNK activation, common signaling pathways activated after NOD1/2 signaling (93). Future studies will determine to what extent effector-mediated NLR activation contributes to colonization and bacterial clearance.

Caspase-1/Caspase-11-deficient mice were found to be hypersusceptible for *C. rodentium* infection, as determined by increased intestinal bacterial loads, colitis, and hyperplasia (94). Both NLRP3 and NLRC4-deficient mice, as well as mice lacking IL-1β and IL-18, showed similar phenotypes, suggesting an important role for the NLRP3/NLRC4/IL-1β/IL-18 axis in the control of *C. rodentium*. In a different study, a similar but stronger phenotype was observed in IL-1R-deficient mice, which mostly succumb to infection within 2 weeks (95). In contrast to what was seen in IL-18-deficient mice, neutralizing this cytokine with antibodies had limited to no effect, implicating IL-1β or IL-1α as the critical cytokines that mediated protection against *C. rodentium*. IL-1R signaling during *C. rodentium* infection led to IFN-γ and IL-6 production in the colon, which mediated epithelial repair and maintained barrier function. While bacterial loads remained the same, more bacteria disseminated to the liver in the absence of these cytokines. Like *Salmonella* and most other Gram-negative bacteria, *C. rodentium* is able to activate the non-canonical Caspase-11/NLRP3 inflammasome in cultured bone-marrow macrophages, which occurred in a T3SS-independent (31, 94). The activation of the Caspase-11/NLRP3 non-canonical inflammasome during infection was evident when examining Caspase-11- and TRIF-deficient mice, which were both more susceptible for *C. rodentium* infection (96). Interestingly, while NLRC4 seems to be activated by *C. rodentium in vivo*, bone-marrow macrophages did not sense the T3SS of *C. rodentium* during *in vitro* studies. Whether this is due to tightly regulated T3SS expression or host cell tropism/specificity remains to be determined.

### Helicobacter pylori

The Gram-negative bacterium *Helicobacter pylori* (*H*. *pylori*) colonizes the gastric mucosa of ~50% of the world’s population, although substantial variation exists between countries (97). The majority of people infected by *H*. *pylori* do not show any symptoms, despite local chronic inflammatory responses induced by the bacterium. However, in a subset of patients, this inflammatory response drives the formation of gastric or duodenal ulcers that can lead to the development of mucosa-associated lymphoid tissue lymphomas and gastric adenocarcinomas (98–100).

In order to survive in the challenging gastric niche and enable persistent colonization, *H*. *pylori* is highly optimized to evade host antimicrobial strategies. For instance, after ingestion *H*. *pylori* secretes urease, which increases the gastric pH and reduces mucus viscosity, enabling rapid penetration of the gastric mucus layer and colonization in close proximity to the pH neutral epithelial cells (99, 101). Also, *H. pylori* expresses a modified LPS and flagellin to evade the recognition of TLR4 and TLR5, and has adopted several mechanisms to counteract the effects of host-produced reactive oxygen species (102, 103). Finally, the secreted pore-forming toxin VacA induces epithelial cell apoptosis and inhibits T-cell activation and proliferation (104). In contrast to immune evasion, a subset of *H. pylori* strains also actively induces inflammatory responses by means of the T4SS-mediated delivery of the effector protein CagA. CagA modifies multiple intracellular signaling pathways of host cells and is linked to the development of gastric cancer.

While immune evasion appears to be an important part of the *H. pylori* life cycle, genetic association studies revealed that mutations in NOD1, NOD2, and IL-1β may be associated with increased risk for the development of gastric cancer, suggesting that NLRs play a role in controlling *H. pylori* during human infection (105–107). In addition, several NLR family members have been shown to sense the bacterium and impact on infection or colonization, both in *in vitro* cell culture and *in vivo* murine models. In a manner analogous to the “leakage” of flagellin through the T3SS in *Salmonella*, peptidoglycan fragments were found to enter the host cytosol through the T4SS, where they were subsequently sensed by NOD1 and initiated NF-κB-mediated inflammatory responses (108). NOD1 activation was also observed by peptidoglycan present in secreted bacterial outer membrane vesicles that were taken up by host cells (109). *H. pylori* was shown to induce the secretion of IL-1β and IL-18 both *in vitro* and *in vivo* (110, 111). As compared to wild-type mice, Caspase-1/Caspase-11-deficient mice showed decreased numbers of *Helicobacter* in the stomach, higher expression of IL-17, and aggravated gastric immunopathology, which was phenocopied by IL-18 and IL-18R, but not IL-1R deficient mice. Loss of IL-18 signaling in dendritic cells was subsequently shown to result in reduced levels of regulatory T-cells and stronger T-cell-mediated antibacterial responses (110, 112). In contrast, different groups reported that Caspase-1/Caspase-11, ASC, IL-1β, and IL-1R-deficient mice were impaired in the clearance of *H. pylori* from the stomach, displayed decreased gastritis and lower levels of IL-1β and IL-18 (111, 113). While the cause of the discrepancies between these different reports is currently unknown, it appears that *H. pylori* strives for the ideal level of inflammasome activation: enough IL-18 and IL-1β to induce regulatory T-cells and decrease gastric acid production, respectively (114), but not so much IL-1β as to lead to T-cell mediated clearance. The nature of the inflammasome NLR that is activated by *Helicobacter* remains unclear. While in cultured dendritic cells NLRP3 was crucial for IL-1β secretion in a T4SS-dependent/CagA-VacA-independent manner, this NLR did not play a role during murine infection.

## The Intestinal Microbiota and NLR-Mediated Disorders

The intestinal microbiota is predicted to consist of ~100 different bacterial species per person, and displays great variability between individuals (115). Alterations in the composition of the microbiota have been shown to dramatically impact disease susceptibility and progression. Therefore, controlling and (re)shaping the “healthy” microbiota is a crucial function of the intestinal immune system. The role of NLRs in this process is only beginning to be unraveled.

Lack of appropriate immunological control may switch a healthy microbiota into a pathogenic one, as exemplified by mice lacking NLRP6. NLRP6-deficient mice show increased levels of intestinal inflammation during DSS-induced colitis and develop more severe colorectal cancer in a model of colitis-dependent tumorigenesis (36, 39, 40, 116). A potential mechanism was provided by the finding that NLRP6 acts as a negative regulator of NF-κB and MAPK activation, and reduces the levels of cytokines and chemokines during infections with intestinal pathogens or epithelial barrier breach as observed during experimental models of colitis (38). More severe and prolonged inflammation in NLRP6-deficient mice results in increased levels of intestinal epithelial proliferation and increased tissue repair, which was shown to be CCL5 and IL-6 dependent (36, 39, 40, 116). The actions of NLRP6 do not seem limited to infectious or damaging episodes, as NLRP6-deficient mice already display continuous low level inflammation in the steady state, suggesting an interaction with the microbiota (36). 16S rRNA sequencing analysis of the microbiota revealed that NLRP6-deficient mice harbor a dysbiotic, colitogenic microbiota that showed a high relative abundance of *Prevotellaceae* species, that was transmissible to wild-type control mice. Similarly, lack of NLRP6-mediated control of the microbiota induced non-alcoholic fatty liver disease and obesity in mice and increased colorectal cancer, all of which were transmissible through microbiota transfer to wild-type mice (116, 117).

NLRP12 and NLRP6 may play similar roles in the control of intestinal homeostasis. Like NLRP6, NLRP12-deficiency leads to uncontrolled NF-κB signaling and subsequent inflammation and intestinal cell proliferation. Although extensive analysis of the composition of the intestinal microbiota of NLRP12-deficient mice has not been reported, the lack of this NLR might have major effects on the microbiota, either directly through sensing microbial products, or indirectly through the induction of an inflammatory environment via NF-κB dysregulation.

While systemic peptidoglycan from the intestinal microbiota was shown to boost the development of the intestinal immune system and prime immune responses via NOD1 in the bone-marrow in mice (115, 118), NOD1, like NOD2, does not dramatically influence the composition of the microbiota under homeostatic conditions (119). However, during DSS-induced colitis, the murine model of inflammatory bowel disease (IBD) that is driven by the microbiota, NOD1/NOD2-deficiency led to greater susceptibility to colitis (120). Similarly, mutations in NOD2 and NOD1 in humans are associated with susceptibility to Crohn’s disease and IBD, respectively (121–123). The role of NLRP3 in controlling the microbiota has been rather controversial. Initially, NLRP3 was reported to have a key role in protecting intestinal homeostasis, as NLRP3-deficient mice were shown to have an altered microbiota and displayed increased susceptibility to DSS-colitis (124, 125) and tumorigenesis (126). However, NLRP3-deficiency led to resistance to DSS-colitis in a different study (127). As the DSS-colitis model is highly dependent on the microbiota, differential compositions of the microbiota may explain the varying outcomes in these studies. Indeed, co-housing NLRP3-deficient mice with wild-type mice, which equalized the intestinal microbiota, also equalized the inflammatory responses and disease in both mice (128). In humans, the role of NLRP3 in Crohn’s is equally confusing; polymorphisms associated with NLRP3 were shown to contribute to susceptibility to Crohn’s disease (129, 130), but did not replicate in a separate study (131). More detailed investigation of the interactions between specific members of the microbiota and NLRs may provide deeper insights in the function of NLRs in controlling and shaping the microbiota in health and disease.

## Concluding Remarks

Nod-like receptors are crucial components of the intestinal innate immune system, controlling both the commensal microbiota as well as enteropathogenic bacterial infections. While a growing body of scientific evidence now provides clear insight into the role of NLRs in controlling intestinal bacteria, several conflicting reports highlight the importance of precisely controlling experimental conditions like bacterial growth phase and the intestinal microbiota between wild-type and NLR-deficient mice. Several key questions still remain unanswered, suck as the nature of the ligands for NLRP6 and NLRP12, the interplay between NLRs and adaptive immunity in the intestine, the potential role for other NLRs like NLRP7 (which senses bacterial lipopeptides in human cells), NLRP10 (which controls adaptive immune responses), and NLRC3 (which down-regulates NF-κB), and the role of NLRs in human diseases. Future research will undoubtedly shed more light on these interesting new subjects.

## Conflict of Interest Statement

The authors declare that the research was conducted in the absence of any commercial or financial relationships that could be construed as a potential conflict of interest.
